# Hearing aid and Koebner phenomenon

**DOI:** 10.1002/ccr3.2762

**Published:** 2020-03-06

**Authors:** Jérôme R. Lechien, Christel Scheers, Mihaela Horoi, Marie‐Paule Thill

**Affiliations:** ^1^ Department of Otolaryngology, Head and Neck Surgery Faculty of Medicine CHU Saint‐Pierre Université Libre de Bruxelles (ULB) Brussels Belgium; ^2^ Laboratory of Anatomy and Cell Biology Faculty of Medicine Research Institute for Health Sciences and Technology University of Mons (UMONS) Mons Belgium; ^3^ Department of Dermatology Faculty of Medicine CHU Saint‐Pierre Université Libre de Bruxelles (ULB) Brussels Belgium

**Keywords:** aid, dermatitis, hearing, Koebner, skin

## Abstract

Koebner phenomenon may be related to frictions of a hearing aid on the external ear and retroauricular skin.

## Graphical abstract

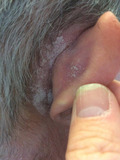

## 1 INTRODUCTION

Koebner phenomenon is a nonspecific cutaneous stimulus eliciting a disease skin reaction. This phenomenon may usually be associated with the development of some skin diseases such as psoriasis, lichen planus, and vasculitis. We report a case of Koebner reaction related to frictions of a hearing aid.

Koebner phenomenon is defined as a nonspecific cutaneous stimulus eliciting a disease skin reaction.^1^ The nature of the underlying skin lesions varies and includes excoriations, frictions, thermal injury, surgical incisions, and scars. Thus, this rare phenomenon may usually be associated with the development of some skin diseases such as psoriasis, lichen planus, vitiligo, sarcoidosis, and vasculitis.^1^, ^2^ In this paper, we report a case of Koebner reaction related to frictions of a hearing aid on the external ear and the retroauricular skin.

## 2 CASE REPORT

**Figure 1:**
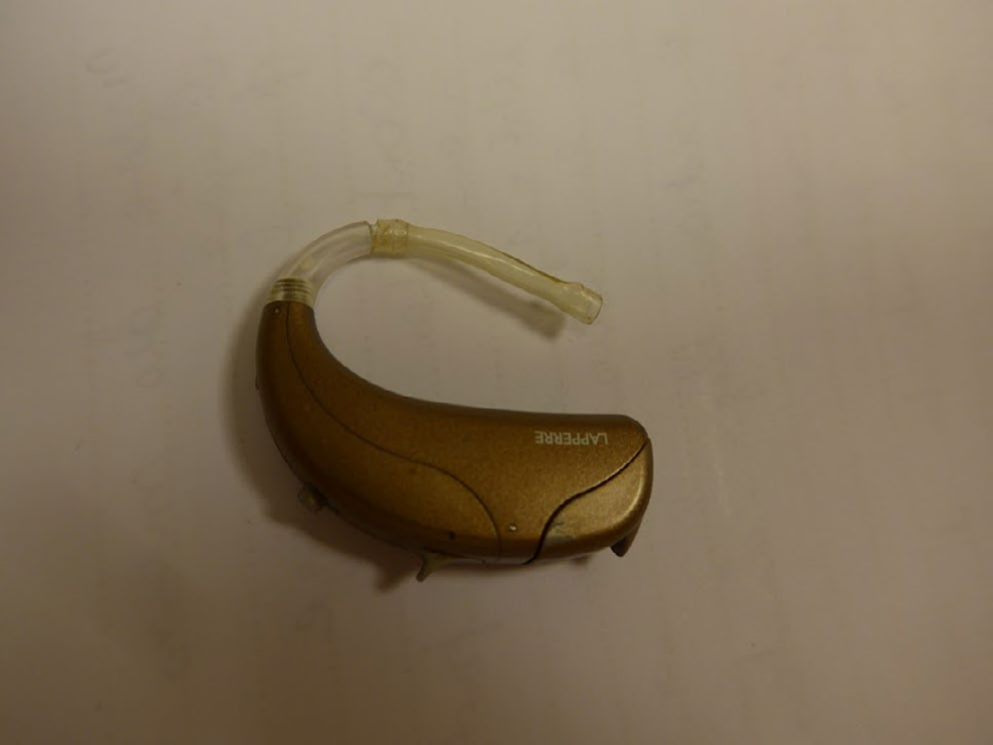
Hearing aid

**Figure 2:**
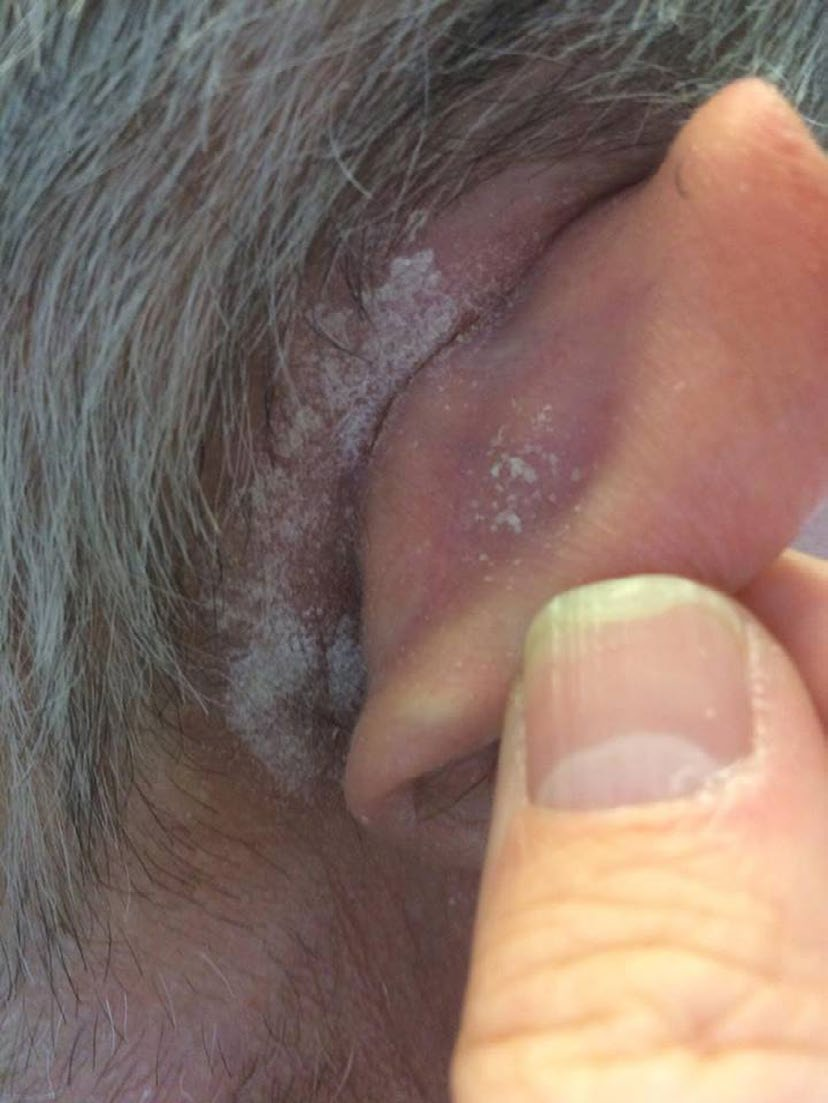
Hyperkeratotic and desquamatory lesions on retroauricular area

A 62‐year‐old man presented in the Department of Otolaryngology—Head and Neck Surgery with a 3‐month history of bilateral retroauricular pruritus. Medical history included presbyacusis and the use of classical hearing aids since 8 years. The patient had a replacement of the hearing aids 3 months ago. Audiologist prescribed a new model with a larger behind‐the‐ear structure (Figure 1). The lesions emerged as hyperkeratotic and desquamatory plaques localized only on the retroauricular part of the scalp (Figure 2). Lesions emerged a few weeks after the first use of the new hearing aids. There was no reaction in the external ear canal or on the ear pinna. The behind‐the‐ear structure was composed of grilamid TR XE 4238 that is a 20% glass fiber reinforced amorphous polyamide, based on aliphatic and cycloaliphatic blocks. The discomfort from the hearing aids and not being able to wear them impacted his quality of life. To exclude contact allergy, patch tests were performed with the European baseline series, cosmetic, pharmaceutical, epoxy, and acrylate series. Readings at 48 and 72 hours were all negative. Patch test with scraped samples of the earplug and behind‐the‐ear structure components did not report any positive reaction. Finally, the complete behind‐the‐ear device was also tested (pasted as a patch) on the pectoral skin but there was no skin reaction. The biopsy supported the diagnosis of a seborrheic lesion (also called sebopsoriasis) secondary to a Koebner reaction since psoriasiform hyperplasia, minimal spongiosis, and scale crusts in a folliculocentric distribution were found. The lack of other similar lesions in the patient also supports this diagnosis. The patient was treated by a corticosteroid ointment for 2 months. After 2 weeks, patient spontaneously interrupted the treatment and used back the hearing aids leading to a recurrence of the lesions. A new model of hearing aids with a smaller behind‐the‐ear structure was prescribed. No recurrence occurred.

## 3 DISCUSSION

Koebner phenomenon is usually found as new skin lesion located on injured or healthy skin.^3^ Since the first case that was reported in 1877,^4^ only a few cases were described in head and neck region.^2^, ^5^ With regard to the etiology, some skin diseases, lacerations, surgical incisions, needle scarification, skin excoriation, or friction are known to be the start point of the disease.^2^, ^3^ In the present case, the behind‐the‐ear structure of the hearing aid caused recurring skin friction both in the retroauricular and ear pinna regions, leading to the development of a seborrheic lesion. Pathogenesis remains uncertain, and several mechanisms have been proposed including immune, vascular, and growth factor–mediated mechanisms.^3^ Koebner reaction is clinically characterized by psoriasis or nonspecific seborrheic lesions underlying pruritus, pain, and itching.^6^

The main suspected diagnosis of this kind of retroauricular lesion remains the allergic dermatitis. Indeed, a dozen cases of allergic reactions related to hearing aid material have been reported in the literature. The most common allergic molecules are methyl methacrylate and polyethylene glycol, which compose the earplug mold or the behind‐the‐ear material. Therefore, patch test is the main additional examination for the diagnosis. In our patient, we performed a classical patch test, which was negative. A second patch test was realized with scraped behind‐the‐ear material applied to the skin, the result was negative. In the case of a second negative result, a biopsy can be indicated to get a final diagnosis.^3^ The differential diagnoses of nonallergic dermatitis in the ear area include dermographism and psoriasis,^3^ which may be caused by skin frictions. In the present case, the diagnosis was supported by the lack of reaction to the patch tests and the nonspecificity of histopathological findings reporting psoriasiform hyperplasia and minimal spongiosis.^6^

## 4 CONCLUSION

The koebnerization of the retroauricular skin related to hearing aids is a very rare cause of dermatitis and one of the main differential diagnoses of contact allergic dermatitis. In both cases, the treatment includes the administration of a corticosteroid ointment. This rare skin reaction may occur at any time of life, and the management needs the collaboration between otolaryngologist and dermatologist to provide an adequate treatment to the patient.

## CONFLICT OF INTEREST

The authors have no conflict of interest.

## AUTHOR CONTRIBUTIONS

JRL: wrote the paper, analyzed the case, conducted literature review, and performed the research. MH and CS: analyzed the case and wrote the part “case report.” MPT: corrected a large part of the paper (clinical arguments and English language).
